# Olive Oil Components as Novel Antioxidants in Neuroblastoma Treatment: Exploring the Therapeutic Potential of Oleuropein and Hydroxytyrosol

**DOI:** 10.3390/nu16060818

**Published:** 2024-03-13

**Authors:** Marta Gonçalves, Anna Aiello, María Rodríguez-Pérez, Giulia Accardi, Emma Burgos-Ramos, Paula Silva

**Affiliations:** 1 Laboratory of Histology and Embryology, Department of Microscopy, School of Medicine and Biomedical Sciences (ICBAS), University of Porto (U.Porto), 4050-313 Porto, Portugal; martamariacg2002@gmail.com; 2 Laboratory of Immunopathology and Immunosenescence, Department of Biomedicine, Neuroscience and Advanced Diagnostics, University of Palermo, 90133 Palermo, Italy; anna.aiello@unipa.it (A.A.); giulia.accardi@unipa.it (G.A.); 3 Biochemistry Area, Faculty of Environmental Sciences and Biochemistry, University of Castilla-La Mancha, Avenue Carlos III s/n, 45071 Toledo, Spain; maria.rodriguezperez@uclm.es (M.R.-P.); emma.burgos@uclm.es (E.B.-R.); 4 iNOVA Media Lab, ICNOVA-NOVA Institute of Communication, NOVA School of Social Sciences and Humanities, Universidade NOVA de Lisboa, 1069-061 Lisbon, Portugal

**Keywords:** neuroblastoma, Mediterranean diet, olive oil, phenolic compounds, oleuropein, hydroxytyrosol, antioxidants

## Abstract

In this review, we explored the therapeutic potential of oleuropein (OLE) and hydroxytyrosol (HT) in the treatment of neuroblastoma (NB). NB is an extracranial tumour that predominantly affects children aged between 17 and 18 months. Recurrence and drug resistance have emerged as the biggest challenges when treating NB, leading to a crucial need for new therapeutic approaches. Food of the Mediterranean Diet (MD) presents several health benefits, including that of cancer treatment. In this review, we emphasised olive oil since it is one of the main liquid ingredients of the MD. OLE is the principal phenolic compound that constitutes olive oil and is hydrolysed to produce HT. Considering that tumour cells produce increased amounts of reactive oxygen species, this review highlights the antioxidant properties of OLE and HT and how they could result in increased cellular antioxidant defences and reduced oxidative damage in NB cells. Moreover, we highlight that these phenolic compounds lead to apoptosis and cell cycle arrest, reduce the side effects caused by conventional treatments, and activate tumours that become dormant as a resistance mechanism. Future research should explore the effects of these compounds and other antioxidants on the treatment of NB in vivo.

## 1. Introduction

### 1.1. Brief Introduction to Neuroblastoma

Neuroblastoma (NB) is a tumour of the autonomous nervous system, specifically the postganglionic sympathetic nervous system. NB primarily affects children aged 17–18 months and originates in the nerve tissue of the adrenal glands, neck, chest, or spinal cord [1,2]. It is the most common extracranial tumour in childhood [3]. Most cases are diagnosed before the fifth year of life, and older children usually have more aggressive and resistant tumours [2,4,5]. Epidemiological studies have shown that NB accounts for 6–10% of all childhood cancers, with an incidence rate of 10.2 per million in children under 15 years of age and a slight male predominance [6,7]. In 2022, the age-standardised incidence rate per million on NB was 8.2, while the value was only 0.11 for peripheral nervous tumours [8]. This disease can exhibit very distinct clinical presentations, varying from a painless abdominal mass to tumours with local invasion that cause critical illness [2,4]. However, the primary tumour location is usually the abdomen, with an origin in the adrenal medulla [1]. Current treatments for high-risk NB involve a combination of chemotherapy, surgical removal, high-dose chemotherapy with autologous stem cell rescue, and radiotherapy. The challenge in the treatment of NB is that of the tendency to relapse and develop drug resistance [1].

### 1.2. Importance of Exploring Natural Compounds in Cancer Treatment

Cancer is a leading cause of mortality worldwide. A primary challenge in cancer therapeutics is that of the transformation of aggressive tumours into chronic illnesses [9]. Over the years, research in this field has evolved, and therapy currently extends beyond conventional treatments such as radiotherapy, chemotherapy, and surgical removal [10]. The escalating cancer rates highlight the crucial need to explore natural compounds for treatment that present themselves as an interesting option for prevention or in conjunction with pharmacological or conventional therapies [9,10]. Furthermore, the usage of natural products is a fundamental approach since they present accessibility and reduced cytotoxicity [10]. These compounds can originate from plants, animals, marine organisms, or microorganisms, and they target molecules found both in normal and tumour cells or molecules that only appear in cancer cell pathways [11,12].

Most drugs used in cancer treatment are only approved for adults as paediatric cancers are a limited area for investment [12]. However, several natural compounds have been the subject of research, specifically for NB [13]. For example, honokiol, obtained from several species of Magnolia, was found to inhibit the production of molecules associated with tumour progression and drug resistance in many types of cancers. In NB, it induces autophagic apoptosis through a tumour protein P53 (p53)-dependent pathway [14,15]. Another interesting example is that of products derived from garlic (*Allium sativum*), which include alliin that originates from cutting, crushing, or grounding garlic and reacts with the enzyme alliinase to form allicin. In NB cell lines, allicin reduced cell division and viability [16]. Moreover, foods from the MD present an interesting option for sources of natural compounds. For instance, in NB cells, an antioxidant that is present in red wine named resveratrol induced cell cycle arrest and apoptosis, and these effects were translated to mice, where the compound presented anticancer effects [17]. Another interesting example is that of lycopene, which is a pigment that is generally present in tomatoes and red fruits and has antioxidant properties, which are fundamental in protecting NB cells against oxidative damage and ER stress-induced damage [18]. In fact, the integration of these natural substances with established chemotherapy protocols offers a promising approach to enhance treatment efficacy and mitigate side effects, which are particularly relevant in paediatric cancer treatment [19].

### 1.3. Significance of Olive Oil in the Mediterranean Diet

In 2010, UNESCO recognised the Mediterranean Diet (MD) as an intangible cultural heritage of humanity [20]. The term MD was first introduced in 1960 by Ancel Keys, who considered it a diet with low levels of saturated lipids and the ability to diminish cholesterol levels in the blood, although the concept itself is thought to date back several millennia [21,22]. Years later, in 1995, Willet et al. described the MD as being a dietary regimen typical of certain regions of Italy and Greece with notably high life expectancy and low rates of diet-associated diseases [23]. Since the Mediterranean encompasses numerous regions, the cultures of different countries are seen in this diet, as well as a wide variety of ingredients with abundant antioxidants and anti-inflammatory nutrients. Fundamentally, the MD consists of an elevated consumption of vegetables, fruits, legumes, unrefined cereals, and nuts. The total fat intake must be lower than 30%, and the saturated fat intake must be even lower, not reaching 10%. The consumption of alcohol should be moderate, as should the intake of animal protein. Regarding energy content, recent descriptions account for around 2000 kcal/day, with 37% of the total calories accounted for corresponding to fat [21,22]. There are several factors that may have influenced the food elements present in this diet, which elucidate why it has been challenging to precisely define this dietary regimen [21]. General descriptions of the MD usually lack specific details such as proportions. However, certain habits, such as adding olive oil to vegetables and legumes to make them more pleasurable or eating fruit as dessert, were stated earlier by some authors. The description of the number of servings allowed for the design of a diet pyramid with different versions [24]. In 2011, the MD Foundation presented a new revised pyramid presentation, with the purpose of sharing a representation of the MD pyramid within the Mediterranean Region [20].

**Figure 1:**
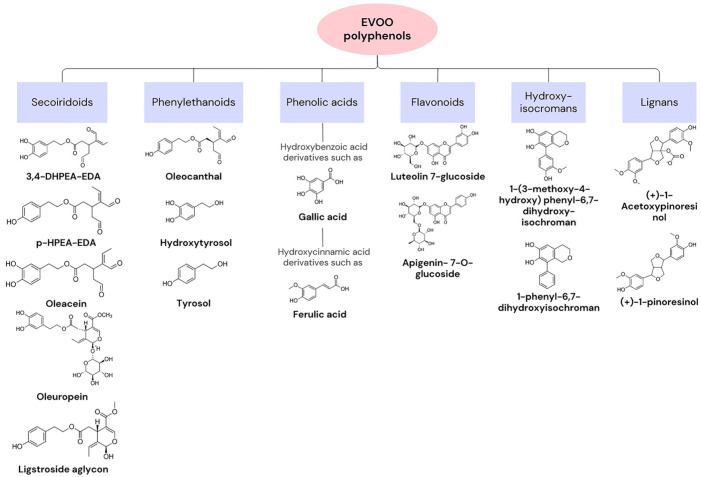
Categories of extra virgin olive oil (EVOO) polyphenols and examples with chemical structures.

Olive oil is the main source of fat in the MD. In Ancient Greece, the olive tree was an essential symbol, and this component was used in medicine and religion, as well as in nutrition [23]. The composition of olive oil varies based on factors such as the origin of the olive tree or climatic conditions. In general, approximately 75% of its content consists of oleic acid (C18:1, n-9), a monounsaturated fatty acid (MUFA). It exhibits low percentages of saturated and polyunsaturated fatty acids [25]. In fact, the positive health effects associated with olive oil are linked to the consumption of extra virgin olive oil (EVOO), which contains high levels of the MUFA and 1–2% highly bioactive compounds. Regarding the MUFA, the existence of a single double bond allows for a higher resistance to oxidation and provides antioxidant properties. Bioactive compounds can be categorised into two groups based on their extraction process. The first consists of unsaponifiable compounds, such as squalene, sitosterols, and pigments, and corresponds to the fraction extracted with solvents after saponification of the oil. The second group consists of hydrophilic compounds, including tocopherol and phenolic compounds [26]. Tocopherol presents high vitamin E activity. Vitamin E is an antioxidant vitamin, with described preventive effects for cancer and cardiovascular diseases. Phenolic compounds, such as oleuropein (OLE), hydroxytyrosol (HT), and tyrosol (Tyr), may act as antioxidants in the human body because they possess a hydroxyl group with that function. These compounds are synthesised from tyrosine or phenylalanine and give a bitter and pungent taste to olive oil since they have a complex interaction with the taste buds [21,25,27]. Figure 1 presents examples of polyphenols that can be found in EVOO, as well as their chemical structure [28].

Targeting aging processes, such as the reduction of telomers, changes in epigenetic patterns, cell senescence, and genomic instability, is one of the numerous abilities of olive oil in preventing disease [29]. These hallmarks of aging are often caused by reactive oxygen species (ROS), which, at elevated concentrations, cause damage to biomolecules such as DNA or proteins. The main production site of ROS is the mitochondria through the reduction of O_2_ to H_2_O during oxidative phosphorylation. As previously mentioned, the phenolic compounds in olive oil have antioxidant properties, allowing for the repression of damage caused by free radicals [21]. Moreover, olive oil has been associated with an increase in longevity and therapeutic health benefits for a variety of diseases. For example, in cardiovascular diseases, it lowers the plasma levels of the low-density lipoprotein (LDL) and increases the levels of the high-density lipoprotein (HLD), diminishing the risk of problems. This fundamental ingredient of the MD reduces the risk of cancer as well as inflammatory and autoimmune diseases [30]. The consumption of EVOO has been studied for its potential in the prevention or treatment of cancer. For instance, HT has demonstrated potent antiproliferative effects against human colon adenocarcinoma cells, suggesting a potential role in cancer treatment and prevention [31]. Moreover, the MD has been linked to a reduced incidence of breast cancer, highlighting its preventive potential [32]. Studies have also shown that EVOO phenols can suppress the migration and invasion of bladder cancer cells, providing insight into the potential of EVOO in cancer management [33]. Olive oil compounds have been found to inhibit vascular endothelial growth factor receptor-2 phosphorylation, which plays a crucial role in tumour angiogenesis [34]. Furthermore, phenols from EVOO have been shown to block cell cycle progression and influence the toxicity of chemotherapeutic drugs in bladder cancer cells [35]. The nutrigenomic approach suggests that different EVOO cultivars, owing to their varied phenolic content, could impact cancer prevention and treatment differently [36]. In summary, scientific evidence supports the potential of EVOO, particularly its phenolic compounds, in preventing and treating certain types of cancer. However, it is important to note that most of these studies focused on adult cancers and in vitro or in vivo models, so further research on paediatric cancers is needed.

### 1.4. Rationale for Studying Oleuropein and Hydroxytyrosol in Neuroblastoma Treatment within the Context of Mediterranean Diet Antioxidants

The MD, which is rich in antioxidants such as OLE and HT, has generated considerable interest for its potential use in cancer treatment. These compounds have gained scientific attention owing to their numerous health benefits including significant antioxidant activity, which is essential for combating the oxidative stress associated with diseases such as cancer. OLE has been identified for its blood pressure-lowering effect and diverse therapeutic potentials, such as cardioprotective, anti-inflammatory, anti-cancer, and neuroprotective functions [37]. This versatility is attributed to its ability to interact with the key molecular pathways involved in disease progression. OLE is the principal phenolic compound in olive oil and is present in distinct parts of the olive. As a member of the secoiridoid family, it hydrolyses to produce HT and 2-(3,4-dihydroxyphenyl)-ethanol. The concentration of OLE is higher during the early stages of fruit development, and esterases transform it into glycosylated derivates [38,39].

In fact, the neuroprotective effects of OLE have been observed through its capability of inducing apoptosis and autophagy and deactivating microglia cells and astrocytes to avoid the unnecessary release of proinflammatory cytokines, thus preventing neuroinflammation. This accounts for the correlation between the consumption of OLE and a decreased risk of developing Alzheimer disease, depression, and other neural disorders [40]. Both OLE and its metabolite, HT, have shown remarkable anticancer activity in different tumours, such as colorectal, breast, bladder, blood, brain, gastric, hepatic, skin, prostate, lung, cervical, and thyroid cancers. Furthermore, these compounds can cross the blood–brain barrier and do not possess toxic effects, allowing them to be candidates for the treatment of different neoplasms [38].

With the tendency to develop drug resistance, the search for alternative therapies for NB has emerged. Considering that OLE and HT have shown remarkable results as potential treatments for different tumours, it is important to explore the potential of these compounds in the context of NB. Research on these substances is still progressing, and numerous studies have emphasised the need for the more extensive examination of their mechanisms of action, particularly in the context of cancer treatment. The encouraging outcomes obtained in preclinical studies offer a strong basis for further enquiry, especially in paediatric oncology where safer and less toxic treatment alternatives are consistently sought.

### 1.5. Objectives of the Review Paper

In this review, we conducted a comprehensive literature search using electronic databases, such as PubMed, Scopus, and Google Scholar, and only peer-reviewed articles written in English were considered. We contemplated the MD and the health benefits of the aliments that constitute it and, more precisely, one of the liquid ingredients in the regimen, that being olive oil. Clearly, we intend to review the currently available information regarding the therapeutic potential of two olive oil components, OLE and HT, specifically for NB, considering that it is prone to recur and tends to develop drug resistance. This review will cover the association between NB and oxidative stress, emphasising the importance of antioxidant interventions in the treatment of tumours and considering OLE and HT as potential therapeutic approaches. We aimed to address the in vitro studies that have been conducted, as well as the possible challenges of utilising OLE and HT in vivo and in clinical trials.

## 2. Neuroblastoma and Oxidative Stress

### 2.1. Definitions and Characteristics of Neuroblastoma

**Figure 2:**
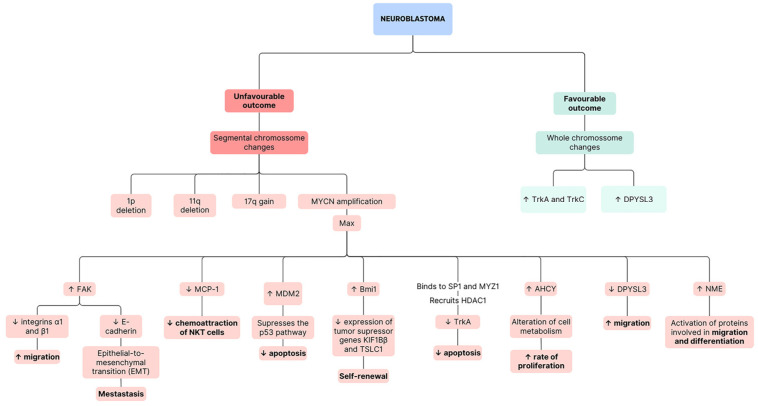
Scheme of pathway alterations in neuroblastoma with favourable and unfavourable outcomes. The bolded text corresponds to the main processes affected, with the upward arrows indicating an increase and the downward arrows denoting a decrease.

As previously stated, NB is the most prevalent extracranial solid tumour in childhood. Disturbance of typical developmental processes at an early stage contributes to tumour initiation, as suggested by the detection of NB in the prenatal stages of life [1]. The evident disparities between clinical presentations have reduced the division of the disease into two groups with genetic differences. While the first one, with a favourable outcome, is usually associated with whole chromosome changes, the second one, with a poor prognosis, is related to segmental chromosome changes, with the most frequent ones being MYCN amplification (2p24), 1p deletion, 11q deletion, and 17q gain (Figure 2) [2,41].

The role of the MYC proto-oncogene family in the biology of both normal and cancerous cells has been extensively investigated. This family, which includes c-MYC, MYCN, and MYCL, is among the most studied groups of proteins in biology. Deregulation of Myc has been implicated in a wide range of cancer types. These genes typically respond to various signals that drive cell proliferation, growth, apoptosis, metabolism, cell-size control, genome integrity, and differentiation. While members of the Myc family share a significant degree of homology, they exhibit slightly different expression patterns. MYCN is a proto-oncogene that encodes information necessary to produce the nuclear protein MycN. This protein contains an N-terminal transactivation domain (Myc box) and a C-terminal domain with a basic helix–loop–helix/leucine zipper (bHLH-LZ) motif. The bHLH-LZ motif mediates DNA binding and interaction with other proteins with the same motif, like Max and Mad. The main function of MycN is to activate the transcription of growth-promoting genes. Nevertheless, to fulfil this role, MycN must dimerise with Max. During the non-dividing phases of the cell cycle, elevated Max levels result in the formation of Max–Max dimers, thereby impeding transcription. In addition, the absence of transcription may be explained by the fact that Mad can compete with MycN for binding [42].

Approximately 50% of patients with NB have metastasis, and evidence shows that MycN regulates the genes associated with metastatic processes. MycN promotes the transcription of focal adhesion kinase (FAK), which is involved in the integrin signalling. Hence, it downregulates integrins α1 and β1, promoting the detachment of the extracellular matrix and, consequently, cell migration. This protein might also suppress E-cadherin, contributing to the epithelial-to-mesenchymal transition. Furthermore, MYCN and p53 may compete for the regulation of FAK levels [43].

On the one hand, MYCN is capable of repressing antigens present in tumour cells to prevent recognition by the immune system. Monocyte chemoattractant protein-1 (MCP-1) is one of the examples of antigens that are repressed, thereby not allowing for the chemoattraction of natural killer T (NKT) cells [44]. On the other hand, MYCN has the dual function of promoting both proliferation and apoptosis depending on the levels of certain apoptotic factors, such as p53 or B-cell lymphoma 2 (BCL2). When NB is initially diagnosed, p53 mutations are rare, indicating that the MYCN gene seems to collaborate with proteins like MDM2 to suppress the p53 pathway and prevent apoptosis. However, as cancer responds to chemotherapy, mutations in p53 become more common, potentially contributing to the development of resistance to therapy [43]. Finally, MYCN targets the promotor of the gene, Bmi1, resulting in its upregulation, which decreases the expression of the tumour suppressor genes KIF1Bβ and TSLC1 (tumour suppressor in lung cancer-1). Overall, this results in the self-renewal of tumour cells [45]. In fact, targeted therapy for MYCN is usually not possible since there are two extended α-helices in the DNA binding domain that do not allow for interaction with small molecules [46].

NB can be hereditary or sporadic in nature [2]. While hereditary tumours have been described as being caused by activating mutations in the kinase tyrosine domain of the oncogene ALK (anaplastic lymphoma kinase), with the gene PHOX2B (paired-like homeobox 2b) also being involved in this process, sporadic NB appears to be associated with single nucleotide polymorphisms (SNPs) in chromosome 6p22, where the gene FLJ22536 is located. This gene encodes a protein that contains an epidermal growth factor (EGF)-like domain [2,47].

In the context of the molecular pathogenesis of NB, Trk is a receptor tyrosine kinase that binds to neurotrophins, which regulate growth and differentiation in normal neural cells. There are three main types of Trk as follows: TrkA (NTRK1), whose ligand is NGF (nerve growth factor); TrkB (NTRK2), which interacts with brain-derived neurotrophic factor (BNDF); and TrkC (NTRK3), which binds to neurotrophin-3. In NB, NGF/Trk signalling plays a crucial role in molecular pathogenesis, and Trk receptors are involved in differentiation, proliferation, survival, invasiveness, angiogenesis, and genomic stability [48,49]. Higher levels of expression of TrkA are generally associated with better outcomes and a lack of structural chromosomal changes. Exposure of NB cells to NGF leads to differentiation, whereas a lack of NGF results in apoptosis [49]. In tumours with MYCN amplification and hence a worse prognosis, the oncogene is able to downregulate the expression of TrkA through binding with the transcription factors SP1 and MYZ1, forming a complex that recruits a histone deacetylase, HDAC1, which induces a repressed chromatin state [50]. In contrast, TrkB is expressed more often in tumours with a worse prognosis and with amplification of MYCN. TrkB/BDNF signalling promotes cell proliferation [49]. Finally, TrkC is commonly expressed in cells that co-express TrkA, indicating its presence in NBs [51].

While genes such as p53, CDKN2A (cyclin-dependent kinase inhibitor 2A), and Ras are commonly aberrant in adult carcinogenesis, they do not present any alterations in primary NB tumours [1]. However, genes AHCY (adenosylhomocysteinase), DPYSL3 (dihydropyrimidinase like 3), and NME1 (NME/NM23 nucleoside diphosphate kinase 1) are involved in this tumour development.

The AHCY gene encodes an enzyme that acts as an inhibitor of methylation into adenosine and L-homocysteine. Overall, AHCY is important for epigenetic reprogramming because it allows for the maintenance of DNA methylation. Specifically, in cancer, it is either a tumour promotor or suppressor depending on the type. In NB cells, its expression is enhanced by MYCN, and it is highly active. In fact, AHCY is a direct target of c-MYC and is associated with a poor prognosis. This gene appears to be involved in the alteration of cell metabolism in the presence of MYCN amplification. AHCY may link glucose to adenosine and L-homocysteine [5]. These cancer cells start relying on oxidative phosphorylation and aerobic glycolysis, with both processes being upregulated to guarantee the efficient production of ATP and the consequent rapid accumulation of biomass and rate of proliferation [52]. This phenomenon can be elucidated by the observation that, while some NB cells exhibit the Warburg effect, certain cell lines with a high content of mitochondria exhibit metabolic flexibility. These cells can shift from glycolysis for oxidative phosphorylation when glucose availability is limited. Overall, MYCN amplification can promote both glycolysis and oxidative phosphorylation in NB [46].

DPYSL3, which is an indicator of a good prognosis, encodes the dihydropyrimidinase-like protein 3 and is a member of the DPYSL gene family, which is normally expressed in the nervous system, particularly in the developmental stages. These proteins have important cellular functions, such as cell migration and differentiation, expansion of axons and dendrites, and axon regeneration. In the context of tumours, their influence on metastatic processes is dependent upon the cancer subtype, with the potential to suppress metastasis. In NB, DPYSL3 physically interacts with F-actin, promoting bundling and inhibiting migration [5]. Nevertheless, MYCN was found to downregulate DPYSL3, possibly through the action of the enhancer of zeste homolog 2 (EZH2), which methylates histones of tumour suppressor genes [53,54].

Lastly, the NME1 gene is located on chromosome 17q, and its gains are associated with poor outcomes for NB. Cells with MYCN amplification usually have high levels of NME1 expression. As mentioned previously, depending on the specific tumour, it may suppress or promote metastasis. In NB, while its function is not entirely clear, it seems important as a histidine kinase, which allows the transfer of a phosphate from a histidine 118 residue to proteins involved in migration and differentiation, resulting in their activation. Moreover, NME1 forms complexes with h-Prune, a member of the phospoesterase superfamily. When comparing the wild-type gene with the one with the S120G mutation, it is noticeable that patients with the mutation present a worse prognosis. This gain-of-function mutation causes the incorrect folding of proteins, affecting their interactions with others [5].

Furthermore, numerous signalling pathways play an essential role in NB, like that of the phosphatidylinositol 3-kinase (PI3K)/protein kinase B (AKT)/mammalian target of rapamycin (mTOR) (PI3K/AKT/mTOR) pathway, which has emerged as a potential target for therapy since it is involved in tumour malignancy. In this pathway, an activated tyrosine kinase receptor interacts with the p85 domain of PI3K, activating the larger catalytic domain p110, which converts phosphatidylinositol 4,5-biphospate (PIP2) to phosphatidylinositol 3,4,5-triphospate (PIP3). PIP3 recruits AKT to the cell membrane where it is phosphorylated by either PDK1 or mTORC2 (mammalian target of rapamycin complex 2). AKT is involved in several processes with significant implications for cancer progression. Specific to NB, AKT leads to the stabilisation of MYCN. While the exact mechanisms that activate this pathway in NB are not yet clear, evidence suggests that ALK mutations might be the answer in certain NBs, as well as some tyrosine kinase receptors and their ligands [55,56].

### 2.2. Oxidative Stress in Neuroblastoma Development and Progression

Oxidative stress can be defined as an excess of ROS, which at normal concentrations play a fundamental role in living organisms by regulating several physiological functions but in excess can easily damage essential biomolecules and cause irreversible damage to the cells [57,58].

ROS production activates redox-sensitive transcription factors that control differentiation, senescence, and transformation into the cancerous state. Oxygen free radicals influence the activity of MAP kinases and several other proteins involved in the cell signalling pathways that control these processes. Hence, redox regulation targeting, for example, the sulfhydryl (RSH) groups of cysteine residues, is fundamental for preventing damage [58].

It is known that cancer cells possess adaptive strategies that guarantee that ROS levels do not result in cell death but rather in proliferation. Oxidative stress is noticeable in both the development and progression of tumours as well as in recurrence. In the early stages of cancer development, pre-neoplasic cells experience high levels of ROS, which lead to the production of antioxidants through the overexpression of genes regulated by nuclear factor erythroid 2-related factor 2 (NRF2) and the redirecting of glucose metabolism to produce nicotinamide adenine dinucleotide phosphate (NADPH), an important molecule involved in oxidative stress. As cancer progresses, ROS stimulate migration and tumour growth, as well as increasing NADPH levels. During metastasis, circulating tumour cells face high levels of ROS, and they also increase NADPH levels to survive in the bloodstream. Finally, during recurrence, cells rely on NRF2-driven gene expression, which helps them to perform β-oxidation instead of glycolysis [57].

The levels of ROS in an animal model of NB have been evaluated, and it has been found that they are higher in the early stages. At 2 weeks, oxidative damage was notorious because there was a fast-replicating NB cell mass. After 6 weeks, the cancerous cells start reducing their ROS concentration to survive, leading to a decrease in intracellular ROS in the progression phase, which promoted the proliferation and progression of the cell cycle [59].

Moreover, data suggest that MYCN amplification, a feature associated with tumours with an unfavourable prognosis, results in increased resistance to oxidative stress [60].

### 2.3. Importance of Antioxidant Interventions in Neuroblastoma Treatment 

Every cell possesses endogenous antioxidant defences (catalase, glutathione peroxidase, and superoxide dismutase, SOD), as well as non-enzymatic endogenous antioxidants (vitamins E and C, coenzyme Q, β-carotene, and glutathione) that are used to mitigate ROS-damaging activity, thereby preventing irreversible modifications to macromolecules [61]. These antioxidants can be classified into two distinct types. While certain ones exert their effects by directly intercepting free radicals through the donation of a hydrogen atom or the transfer of an electron, the others act indirectly on substances that have the ability to promote the formation of free radicals [62].

In cancer treatment, certain chemotherapeutic compounds generate free radicals to eliminate the tumour cells. However, this approach typically results in several adverse effects. Evidence shows that, despite cancer patients experiencing heightened oxidative stress, their antioxidant levels tend to remain low. Notably, some studies have included vitamin C as part of an antioxidant blend administered along with chemotherapy to reduce side effects arising from toxicity. Moreover, there are several clinically approved antioxidants, like amifostine, employed in cancer treatment to protect healthy cells from the adverse effects of therapy [61].

The antioxidant properties of several compounds have been explored over the years in NB treatment. For instance, NB cells treated with N-acetylcysteine (NAC), a derivative of methionine that increases the activity of antioxidant enzymes, have lower proliferation rates and lose their ability to anchor in distinct places to grow colonies. These findings suggest that by inhibiting ROS, NAC decreases the tumorigenicity of NB [63,64]. Furthermore, Molina-Jimenez et al. (2005) demonstrated that fraxetin, a coumarin, protects the cell against rotenone effects by reducing neurotoxic intracellular ROS levels [65]. Another study showed that polyphenols from aqueous extracts of cocoa have showed remarkable potential in the protection of NB cells against oxidative stress [66].

While further research is needed, it is reasonable to assume that antioxidants can have beneficial effects for cancer therapy, particularly in NB treatment, since they are able to balance ROS levels to prevent them from harmful activities and to possibly mitigate the side effects caused by certain conventional treatments.

## 3. Oleuropein and Hydroxytyrosol

### 3.1. Introduction to Oleuropein and Hydroxytyrosol

OLE, the primary phenolic compound found in olives (Oleaceae family), can constitute as much as 14% of their total weight [39]. It was discovered in 1908 by Bourquelot and Vintilesco and consists of three subunits as follows: secoiroid (elenolic acid), polyphenol (HT), and the glucose molecule. OLE formation in olives occurs as part of the secondary metabolism of terpenes. Within this metabolic pathway, the mevalonic acid cycle plays a crucial role, where a branching event gives rise to OLE [67]. In fact, the quantity of this compound in olives decreases with physiological development.

OLE metabolism can occur in the stomach or intestine depending on whether administration occurs through gastro-resistant capsules. When metabolism occurs in the stomach, either the acidic environment or the action of a β-glycosidase cleaves the β-glycosidic bond in OLE originating from the glucose and aglycone moieties. The aglycone transforms into two dialdehydes that are unstable and quickly converted into transposed secoiridoid, a lipophilic compound that under specific conditions such as prolonged exposure to an acidic environment can lose HT or methanol. If OLE is administered through capsules that resist the acidic environment of the stomach, it may reach the intestine unchanged. However, because OLE is a hydrophilic molecule, it cannot be absorbed by the cell membrane [39]. Although no clear evidence has shown that glucose transporters are specifically involved in the uptake of OLE, Hollman et al. (1995) stated that glucose transporters might be involved in the absorption of glycosylated biophenols [68]. Nevertheless, research has shown that in an isolated rat intestine, OLE is poorly absorbed [69].

**Figure 3:**
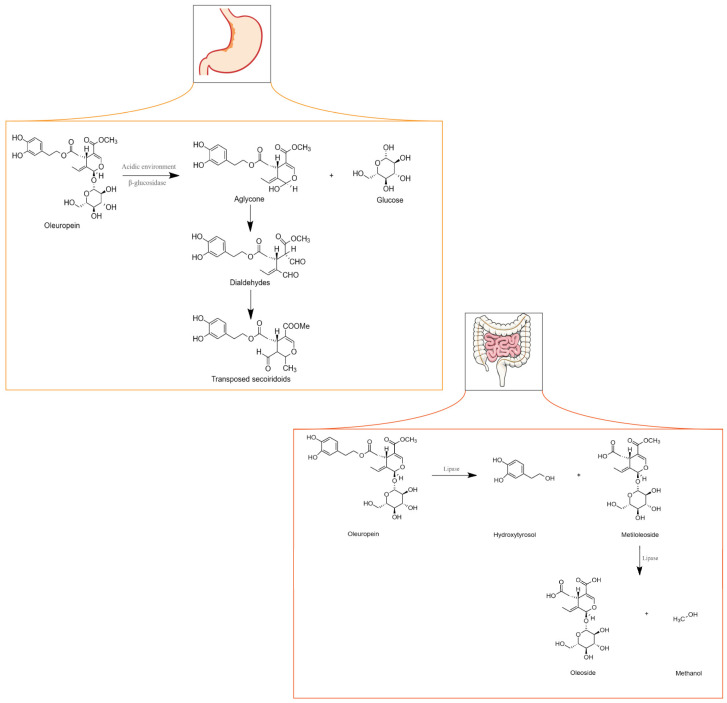
Metabolism of oleuropein in the stomach and intestine. Adapted from [71].

As depicted in Figure 3, within the intestines, OLE undergoes transformation into HT and methyloleoside through lipase activity. Subsequently, another lipase converts the methyloleoside into oleoside and methanol.

OLE exhibits noteworthy versatility and pivotal roles in diverse biological processes. Some examples include its ability to decrease inflammation through the reduction in nuclear factor kappa-light-chain-enhancer (NF-kβ) activation and translocation to the nucleus, thereby resulting in the lack of expression of genes encoding inflammatory mediators. Additionally, it demonstrates neuroprotective activity since it increases the expression of NGF, BDNF, and their receptors TrkA and TrkB [70].

In cancer, OLE inhibits cell growth, motility, and invasiveness. OLE exerts its functions through, among other factors, the inhibition of NF-kβ and hypoxia-inducible factor 1α (HIF-1α), upregulation of pro-apoptotic genes and tumour suppressor miRNAs (miR-125b, miR-16, miR-34a, p53, p21, and TNFRS10B), and a decrease in the expression of histone deacetylases (HDAC) [71]. In prostate cancer, OLE is a pro-oxidant in neoplastic cells and an antioxidant in healthy cells. This results in decreased proliferation and cell death in tumours. Furthermore, OLE and HT contain an aromatic ring similar to that of oestradiol in their molecular structure, suggesting that these natural compounds may compete with oestradiol for binding to the oestrogen receptor (ER). This is particularly important in breast cancer, in which oestradiol stimulates cell growth. These polyphenols might inhibit cell proliferation in a concentration-dependent manner. Some studies have suggested that OLE induces cell death, especially in ER-negative tumours [39]. Figure 3 illustrates the further effects of OLE on distinct tumours.

As previously mentioned, olive oil, under acidic conditions, originates from the polar phenolic compound HT. Nevertheless, HT may also result from the enzymatic breakdown of its respective glycosides. HT is an anti-inflammatory, antiatherogenic, and antithrombotic compound. Furthermore, it is slightly soluble in water as well as in lipids [72]. HT and OLE suppress LDL oxidation. However, while the concentration of the latter decreases with maturation of the olive fruit, the amount of HT increases [38].

Several methods have been developed for HT synthesis. One approach is to use lithium aluminium hydride to reduce the methyl ester of 3,4-hydroxyphenyl acetic acid. The absorption process occurs in the intestine, where the compound enters through bi-directional passive diffusion. HT can be oxidised to a 3,4-dihydroxyphenylacetaldehyde sulphate conjugate, 3,4-dihydroxyphenylacetic acid, glucuronide conjugate, homovanillyl alcohol, and homovanillic acid. Excretion predominantly occurs through the renal route, where the compound is eliminated in urine as glucuronide conjugates. At high concentrations, they may exhibit considerable toxicity [38].

**Figure 4:**
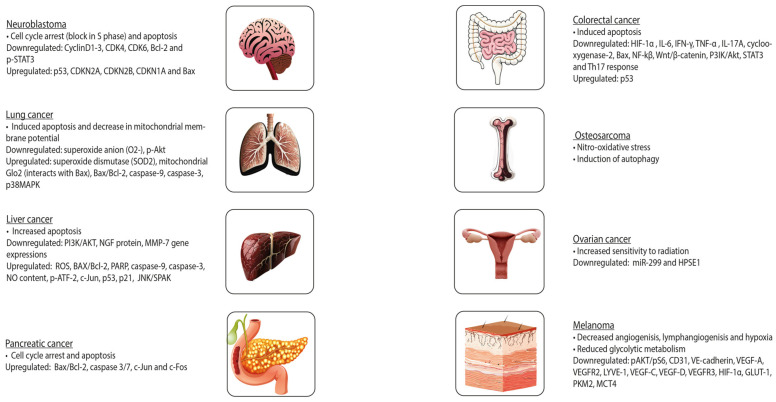
Effects of oleuropein on different tumours.

In fact, previous research has demonstrated that HT can hinder the initiation and progression of tumours, which is in accordance with the described findings for OLE (Figure 4). It can prevent DNA from oxidative damage and induce apoptosis. Furthermore, the addition of sulphur-containing functional groups to HT may reverse resistance to anti-cancer drugs in leukaemia, suggesting that exploring potential beneficial structural modifications of HT might be an interesting approach in this field [73].

### 3.2. Sources and Bioavailability in Mediterranean Diet Foods

Both OLE and HT present low bioavailability [74,75]. They are present in table olives and olive oil. Notwithstanding, yeast produces HT during alcoholic fermentation by metabolising aromatic amino acids.

As previously mentioned, the decline in OLE and the rise in HT levels occurs as olives mature, establishing the latter as the predominant compound in mature olives. In fact, table olive processing results in the existence of three types of olives as follows: green olives, dark olives obtained through oxidation, or natural darkened olives. Green olives undergo lye treatment with a NaOH solution that leads to the conversion of OLE into HT and elenolic acid glucoside. Moreover, yeast and lactic acid bacteria that naturally exist in olives are also capable of metabolising OLE. The formation of olives darkened by oxidation implicates their preservation in brine (concentrated solution of sodium chloride in water) or an acidic solution and then darkening with air under alkaline conditions. The processing method surpasses the impact of both the variety and geographical origin on table olives’ phenolic compound content [76]. Generally, the mean content of OLE in black olives is 72 mg/100 g and 56 mg/100 g in green olives. Regarding HT, the average content is 659 mg/kg in black olives and 556 mg/kg in green olives.

The average OLE content in olive oil ranges from 0.17 mg/100 g in extra virgin oil to less than 1 µg/100 g in virgin varieties. Conversely, the average content of HT in olives is 3.5 mg/kg in virgin olive oils and 7.7 mg/kg in EVOOs. However, the amount of time in storage that precedes the analysis is an important factor to consider in determining the exact concentrations of OLE, HT, and other metabolites. Fresh EVOO presents higher concentrations of oleocanthal and oleacein and a lower quantity of HT and Tyr. Furthermore, cooking implies thermal oxidation that alters the phenolic compound content of olive oil, where HT derivates are among the initially depleted components [76].

### 3.3. Mechanisms of Antioxidant Action

The effectiveness of OLE and HT in providing antioxidant benefits is linked to their relative bioavailability. These phenolic compounds can act in two distinct ways, either by generating stable resonance structures through the scavenging of peroxyl radicals and the breaking of peroxide chain reactions or by averting the cooper sulphate-induced oxidation of LDL. The latter described mechanism consists of metal chelator activity, which is guaranteed since OLE and HT have hydroxyl groups capable of engaging in intramolecular hydrogen bonds with free radicals. The existence of these mechanisms was proved using metal-independent oxidative systems and stable free radicals [27,77]. Furthermore, HT induces the activity of detoxifying enzymes and promotes mitochondrial biogenesis, with both processes being fundamental in protecting against oxidative damage. HT can also regulate an adaptive signalling pathway that is activated in response to ER stress, and it also contributes to improving ER homeostasis [78]. The described antioxidant properties of these phenolic compounds have been proven to be stronger than those of vitamin E or butylated hydroxytoluene [27]. In cancer cells, at higher doses, OLE and HT have demonstrated a pro-oxidant activity, which is correlated with their anti-proliferative effects [78]. This pro-oxidant activity could also be associated with the cytotoxicity of the compounds at these high concentrations [79].

NRF2 can regulate protection against oxidation. In normal situations, Keap1 (Kelch-like ECH-associated protein 1) targets NRF2 and promotes its degradation in the proteasome. In stress-inducing situations, cysteine residues of Keap 1 are oxidized, thereby not allowing for its binding to NRF2 [80]. This results in the activation of NRF2, followed by translocation to the nucleus, where it binds to antioxidant response elements (AREs) and, subsequently, leads to the expression of antioxidant enzymes, such as SOD, c-glutamylcysteine synthetase (c-GCS), glutathione S-transferase (GST), and NADPH quinone oxidoreductase (NQO1). In in vivo studies, at a concentration of 5.0 mg/kg of HT, male mice C57BL/6J showed a decrease in oxidative stress since HT restored NRF2 and the levels of the peroxisome proliferator-activated receptor α (PPAR-α). When a higher concentration of HT was used, there was an increase in the activity of GST in the liver and muscle. In studies where OLE was utilized, there was an increased expression of enzymes regulated by NRF2 [77].

## 4. Potential Effects of Oleuropein and Hydroxytyrosol on Neuroblastoma

### 4.1. In Vitro Studies: Impact on Neuroblastoma Cell Lines

#### 4.1.1. Cell Viability and Proliferation

The effects of OLE and HT on NB have been explored in various cell lines. Considering this, the authors conducted an evaluation of cell viability and proliferation after treatment with these phenolic compounds.

In SH-SY5Y NB cells, at 48 h, using a concentration of 350 µM of OLE, half of the cells had reduced viability (IC50 dose). This was a time- and dose-dependent effect since high concentrations, as previously mentioned, are associated with cytotoxicity [81]. On the other hand, in CHP-134 (chemotherapy and irradiated late response) NB cells, at 48 h of incubation and with an OLE concentration of 1000 µM, the percentage of cell viability was less than 60% [82]. The clear differences in the concentration of OLE required for the achievement of approximately 50% viability between the SH-SY5Y and CHP-134 cells could be attributed to the fact that the CHP-134 cell line corresponds to cells after exposure to chemotherapy or radiation, which potentially leads to higher ROS levels. Consequently, a higher concentration of OLE is required to observe significant effects in the latter cell line.

Lastly, upon the exposure of SH-SY5Y cells to HT, the concentration that affected half of the cells at 48 h was 113.59 µM [83].

Overall, the evidence indicates that both OLE and HT can reduce cell viability and proliferation in a dose-dependent manner, although using OLE implies the need for a higher concentration of the compound to achieve comparable results to those obtained with HT. These results can be explained by the fact that HT is a stronger antioxidant than OLE and are in accordance with previous research conducted on pancreatic cancer cell lines [84].

#### 4.1.2. Induction of Apoptosis and Cell Cycle Arrest

OLE treatment resulted in a higher incidence of apoptotic cells in SH-SY5Y NB cells, accompanied by an increase in the expression of the BAX (Bcl-2-associated X-protein), BAD (BCL2-associated agonist of cell death), BID (BH3-interacting domain death agonist), DFFA (DNA fragmentation factor subunit alpha), Caspases 1, 3, 9, and 8, and the p53 genes, as well as by a decrease in the anti-apoptotic gene Bcl-2. Concerning the cell cycle, OLE led to cell cycle arrest, with the upregulation of several genes being responsible for inhibiting cellular proliferation and tumour growth, such as CDKN2A (cyclin-dependent kinase inhibitor 2A), CDKN2B (cyclin-dependent kinase inhibitor 2B), CDKN1A (cyclin-dependent kinase inhibitor 1A gene), p53, and GADD45A (growth arrest and DNA damage-inducible alpha), as well as the downregulation of genes that play an important role in the regulation of the cell cycle, like CCND1 (cyclin D1), CCND2 (cyclin D2), CCND3 (cyclin D3), CDK4 (cyclin-dependent kinase 4), CDK6 (cyclin-dependent kinase 6), and WEE1 [81]. In CHP-134 NB cells, at 24 h and at an OLE concentration of 500 µM, 12% of the cells underwent apoptosis and 45% underwent necrosis. Moreover, in LAN5 cells, 10% of the cells experienced apoptosis and 3% experienced necrosis [82].

Moreover, an OLE derivative, OLE aglycone (OLEA), showed remarkable anticancer effects in NB cells. OLEA reduces NB cell growth through various intracellular pathways, inducing apoptotic cell death by increasing pro-apoptotic proteins (Bax and p53) and decreasing anti-apoptotic proteins (Bcl-2). OLEA also inhibits the phosphorylation of STAT3, a transcription factor implicated in tumour formation, leading to reduced cell migration and invasion [85].

The administration of HT in SH-SY5Y cells resulted in an apoptotic rate of 79.64%, suggesting a potential use of this compound in cancer treatment [83]. Moreover, in the BE (2)-C NB cell line, HT upregulates protective genes such as p53 and Bcl-2 while downregulating the pro-apoptotic genes Bax and caspase-3. These factors could be important in the future development of therapeutic strategies aiming for the equilibrium between the protection of normal cells and the eradication of tumour cells [86].

In fact, both phenolic compounds demonstrate significant potential in inhibiting tumour growth and decreasing cell survival through the induction of apoptosis and cell cycle arrest.

#### 4.1.3. Reduction of Oxidative Stress

The antioxidant properties of OLE are in part attributed to its ability to bind to metal ions, including copper and iron, inhibiting them from catalysing free radical production reactions. Copper can in fact be responsible for OLE antioxidant actions on tumorous cells, particularly SH-SY5Y cells [87,88].

The modulation of gene expression performed by HT in BE (2)-C cells leads to enhanced cellular antioxidant defences and decreased oxidative damage. Moreover, HT influences the NRF2 pathway, boosting the expression of antioxidant enzymes and activating peroxisome proliferator-activated receptor gamma (PPAR-γ), thereby reducing inflammation and oxidative stress [86].

In summary, OLE and HT present a noteworthy capability of protecting cells against oxidative damage through both anti-apoptotic and antioxidant properties.

### 4.2. Challenges in Utilising Oleuropein and Hydroxytyrosol in In Vivo Studies: Animal Models and Clinical Trials

Polyphenols present poor bioavailability as they are poorly absorbed and rapidly metabolised and excreted in the human body. Evidently, this poses a substantial challenge in conducting in vivo studies since the dose of the compounds delivered to the tumour cells might be low. A solution for this problem is that of a combination with other phenolic compounds or with anti-cancer drugs [89].

Although no in vivo studies have up to now specifically examined the effect of OLE administration on NB, some animal trials showed its anticancer potential. OLE’s capacity to inhibit tumour growth and metastasis was demonstrated in studies using ovariectomized nude mice xenografted with MCF-7 human breast cancer cells. This intervention not only impeded the reduction in body weight but also arrested the dissemination of tumour masses to the lungs [90]. In accordance with previous statements, the combination of OLE with doxorubicin increased the effectiveness in reducing the tumour size after four weeks of treatment by three times. In fact, combination therapy triggered apoptosis in 85% of cancer cells, compared to 40% with OLE and 60% with doxorubicin when both were used alone. This therapy also downregulated oncoproteins related to breast cancer and upregulated the p21WAF1 gene, which inhibits the growth of cancer cells [91]. In a study involving mice exposed to dextran sulphate sodium (DSS) and azoxymethane (AOM), OLE decreased markers of inflammation and reduced colon tumour development. The compound also showed potential in inhibiting the proliferation of neoplastic cells, downregulating Ki-67, upregulating Bax, and inhibiting the activities of NF-kB, Wnt/β-catenin, Akt, and STAT3 (signal transducers and activators of transcription 3) [92]. Additionally, oral OLE administration to male hairless mice prevented ultraviolet B (UVB)-induced skin damage and carcinogenesis, with the highest concentration tested showing the most potent effect. This was associated with the suppression of vascular endothelial growth factor (VEGF), matrix metallopeptidase 2 (MMP-2), matrix metallopeptidase 29 (MMP-9), and matrix metallopeptidase 2 (MMP-13) expression as well as reduced cyclooxygenase-2 (COX-2) levels [93]. Finally, a study of the pre-initiation phase of tumorigenesis in female mice found that OLE delayed the formation of epidermal hyperplasia, a precursor of skin tumours, and decreased oxidative stress markers. Additionally, it increased the levels of the antioxidants glutathione (GSH) and SOD, suggesting antioxidant and apoptotic activities. However, limitations were also noted, such as the short treatment period of 10 weeks and lack of observation for papilloma or carcinoma formation [94].

There is no record of in vivo studies utilising HT in NB. Notwithstanding, the anti-cancer effects of HT in Sprague Dawley rats with experimental mammary tumours have been demonstrated. The phenolic compound is capable of inhibiting cancer cell growth and proliferation, yielding comparable outcomes to those observed with the administration of the anticancer drug doxorubicin. Indeed, HT downregulated Ki-67 and upregulated genes related to apoptosis (Fas or Tnfrsf6, Emp2), cell cycle, cell proliferation and differentiation (Sfrp4, Fhl1, Wisp 2, Mertk, Fgfr2, Cdh13, Smarca2), inflammation (Il6st), and response to stress (Cryab, Gstm2). Furthermore, it decreased the expression of other apoptosis-related genes (Atf3, Gas5, Ier3, Bhlhb2, Sqstm1), cell cycle, proliferation, and either differentiation genes (E2B, Ube2b, Cxcl1, oncogene Jun, Hmgn2) or inflammation genes (Mcpt8, Mcpt9, Mcpt10) [95]. Another study illustrated the effect of HT in mouse pancreatic cancer. In fact, the compound inhibited the proliferation of tumour cells, was able to activate the signal transducers and activators of the transcription 3 (STAT 3)/CCND1 signalling pathway, increased the proportion of M1 macrophages, and enhanced the anti-cancer effects of the anti-CD 47 antibody. Additionally, the combination of HT with plumbagin (PLB) resulted in more cell death compared with HT or PLB alone [96].

Overall, as previously stated, the capability of OLE and HT to suppress tumour growth by inhibiting cell proliferation and inducing apoptosis have been clearly verified in vitro. This efficacy has further translated into in vivo studies that confirmed these outcomes in distinct tumours. Nonetheless, in the context of NB, there is a clear need to expand the current knowledge through the conduction of in vivo investigations.

Problems related to bioavailability, bioaccessibility, and dosage result in few clinical trials with OLE and HT. However, there are some clinical trials aiming to understand the cardiovascular, protective effects of these compounds, as well as their effect in neurodegenerative diseases [75,97]. Furthermore, the European Prospective Investigation into Cancer and Nutrition (EPIC) and the PREvención con DIetaMEDiterránea (PREDIMED) trials have shown that regular consumption of EVOO in the context of the MD diminishes the occurrence of several chronic diseases, including that of cancer. Within the PREDIMED trial, a 68% reduction in the incidence of breast cancer among women that followed a diet supplemented with EVOO was registered [32]. Nevertheless, it is fundamental to recognize that the outcome cannot be attributed to HT or OLE alone since EVOO contains other components [97].

### 4.3. Enhancement of Conventional Cancer Therapies through Antioxidant Intervention

Antioxidants have been established early on as important compounds in cancer prevention. Nevertheless, a concern emerged that they could reduce the effects of treatments such as radiotherapy, which uses ionising radiation to form ROS, eventually leading to cell death. In general, however, the usage of antioxidant supplementation along with conventional therapy brings many advantages. For instance, patients tend to have more treatment tolerance and live longer than those who did not experience supplementation. There are some exceptions to this, in which utilising both antioxidants and conventional therapy is not favourable, including the use of flavonoids with tamoxifen, NAC with doxorubicin, and β-carotene with 5-fluorouracil [98].

According to Khurana et al. (2018), the efficacy of the usage of antioxidants along with chemotherapy in animals has been demonstrated early on. Notwithstanding, this efficacy was challenged in some trials in humans. These early studies were poorly designed, which could explain the inconsistent outcomes. In fact, antioxidants can be either natural or synthetic. Although natural antioxidants are preferable, it is hard to calculate a safe concentration since there is a lack of studies performing safety tests. Hence, the improvement of natural antioxidant databases is of extreme importance. This author further suggests the administration of antioxidants before and after the chemotherapy rather than during treatment [99].

The generation of increased ROS levels in tumour cells by chemotherapeutic agents might lead to dormancy and, consequently, drug resistance. Although the use of antioxidants is controversial, they could suppress oxidant stress caused by chemo-drugs, hence protecting normal cells, and they could also activate a dormant tumour to make it susceptible to chemotherapy. Nevertheless, further studies are needed to address the exact cell signalling mechanisms that are affected by antioxidants in all types of cells and to allow for the establishment of a baseline [100].

## 5. Conclusions

The anticancer potential of OLE and HT has been explored over the years. As part of olive oil, a fundamental ingredient of the MD, it is expected that these compounds present several benefits due to their antioxidant properties. Cancer cells produce an increased amount of ROS that, instead of leading to cell death, help in their proliferation while potentially damaging normal cells. Antioxidant actionhas been investigated over years for cancer treatment since it is not only able to adjust ROS levels to prevent them from harmful activities, reducing the side effects caused by conventional treatments, but also activates tumours that became dormant as a resistance mechanism. In NB, ROS levels are higher in the early stages but decrease in the progression phase to allow tumour cells to proliferate more easily. Aggressive tumours with MYCN amplification tend to be more resistant to oxidative stress due to the increased transcription of glutathione. Despite their low bioavailability, OLE and HT had remarkable effects against the proliferation of NB cells, thereby leading to apoptosis and cell cycle arrest. Moreover, these polyphenols allow for the enhancement of cellular antioxidant defences and a decrease in oxidative damage. The insights present in this review allow for a more comprehensive understanding of the capability of these compounds in NB treatment. The remarkable features of OLE and HT provide a distinctive opportunity to investigate the effects of these and other phytochemical compounds from olive trees, such as oleacin and oleocanthal, in vivo and in further clinical trials, possibly contributing to the resolution of a critical challenge when treating NB—the tendency to relapse and develop drug resistance. In conclusion, it seems imperative to conduct further studies exploring the role of antioxidants in addressing the enduring challenges of treating NB and other cancers.
